# Supplementary material for: Exploring the effectiveness of a COVID-19 contact tracing app using an agent-based model
Source: Sci Rep. 2020 Dec 17;10:22235. doi: 10.1038/s41598-020-79000-y (PMC7746740; doi:10.1038/s41598-020-79000-y)
Supplement: Supplementary file 1 — Supplementary Information. [file 41598_2020_79000_MOESM1_ESM.pdf]

Exploring the effectiveness of a COVID-19 contact tracing app using an agent-based model - Supplementary information

Jonatan Almagor, Stefano Picascia

3rd November 2020

Disease state transition: probabilities, duration, literature sources

Parameter	Description	Value	Reference
α^A	Probability of infected agent of age A of becoming symptomatic	age group: 0-10, 10-20, 20-40, 40-50, 50-70, 70+<br>probability: 2%, 26%, 55%, 62%, 72%, 82%	[1]
δ^A	Probability for symptomatic agent of age A to progress to severe disease	age group: 0-15, 15-40, 40-50, 50-60, 60-70, 70+<br>probability: 2%, 6%, 9%, 13%, 17%, 20%	[2]
$\gamma^{A,G}$	Probability of death for severely ill agent of age A and gender G	age group: 0-15, 15-40, 40-50, 50-60, 60-70, 70+<br>male probability: 0.5%, 3%, 8%, 9%, 16%, 25%, 50%<br>female probabilities by age corresponds and reduced by 20%	[3]
d_{inc}	Incubation period	Value drawn from Gamma distribution (5.1, 1)	[4]
d^A_{asy}, d^A_{mild}	Disease duration of asymptomatic and mild symptomatic agent of age A	age group: 0-40, 40-50, 50-60, 70+<br>mean duration, days: 8, 12, 15, 20.<br>Value drawn from normal distribution with the age group mean and $SD = 0.25 * mean$	[5]
d_{sev}	Duration of severe disease before hospital admission	Gamma distribution (6.5, 0.9)<br>mean = 7 days	[6]
d^A_{hos}	Length of hospital stay	age group: 0-40, 40-50, 50-60, 70+<br>mean duration, days: 8, 12, 15, 20<br>Value drawn from a normal distribution with the age group mean and $SD = 0.25 * mean$	[6]

Table 1: Disease state transition probabilities and duration, with literature reference

Sensitivity analysis

In order to test whether the impact of the CTA on transmission dynamics as demonstrated in our simulation still holds under various assumptions of contact patterns in the population and under different transmission probabilities, we conducted a sensitivity analysis to selected parameters as specified in Table 2. The sensitivity analysis was carried out assuming a social distancing scenario and a testing policy that prioritises symptomatic agents when testing capacity is 1.5% of the population per week, and unlimited. The results are presented in the figures below. In all the figures, the black trajectory represents the value used in the simulations discussed in the main paper.

Parameter	Description	Value in model	Range tested for sensitivity	Figure
β_c	Transmission probability per network contact	0.056	0.028 – 0.084	Fig. 1
p	Percentage of local area population that agents meet in random encounters	0.7%	0.35% – 1.4%	Fig. 2
β_r	Transmission probability per random contact	0.0056	0.0028 – 0.0112	Fig. 3
f	Number of friends that agents meet per encounter	Random draw from 1-10% of agent's ties	Varying no. of friends by: -50% – +100%	Fig. 4

Table 2: Parameters and range of values tested in sensitivity analysis

The diagrams show that, as expected, varying each of the parameters does influence the overall number of infections, however the main model outcome - that higher CTA adoption rates always translate in lower infections - is consistently emerging. The influence of the CTA can be observed by the negative slope in all the figures. The steepness of the slope depends on the degree to which CTA adoption decreases the spread.

In particular, the sensitivity analysis shows that the CTA is more effective in scenarios of higher viral circulation (more contacts or higher infectiousness). At the higher values of all the parameters tested more infections are generated than those in the base model. For these conditions a steeper slope is observed, indicating a higher relative reduction in infections as CTA adoption rates increases. This dynamics can be be explained: in higher transmission conditions

each infectious agent infects more susceptible agents (on average) compared to conditions of low transmission; therefore each infected case who is also a CTA user sends alerts to more agents who were exposed to him and were infected, which in turn reduces their exposure to the population (by self-isolating); this translates into a relatively higher reduction in infections. On the contrary, for low conditions of transmission the viral circulation is reduced overall, and therefore the effect of the CTA is less apparent, because it is activated in less infection occasions.

This effect is particular noteworthy as countries around the world enter the second wave of the pandemic and at the same time are trying to avoid strict lockdown and maintain sections of the economy open, which results in ongoing interactions between people. Under these circumstances high CTA adoption rates in the population combined with sufficient testing capacity can significantly reduce the spread of COVID-19.

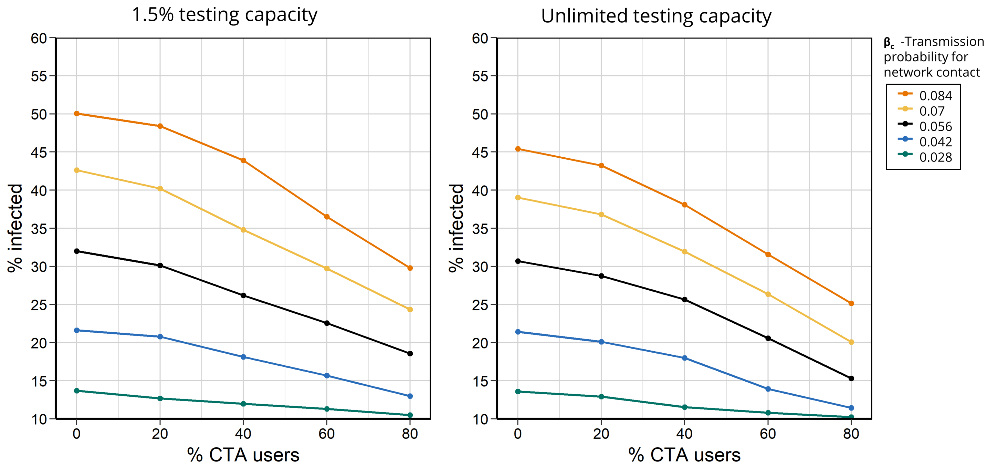

Figure 1: Sensitivity analysis for transmission probability per network contact (friends and work colleagues) (β_c). For each β_c value used in the simulation we present the percentage of the population infected during the course of the epidemic (y-axis) for varying rates of CTA users (x-axis). Values of β_c are presented by lines with unique colours. Black line represents the value used in paper. The slope of the trajectory represent the influence of the CTA; a steeper negative slope represent a larger relative reduction in infections. Scenarios with testing capacity of 1.5% (left plot) and unlimited testing capacity (right plot).

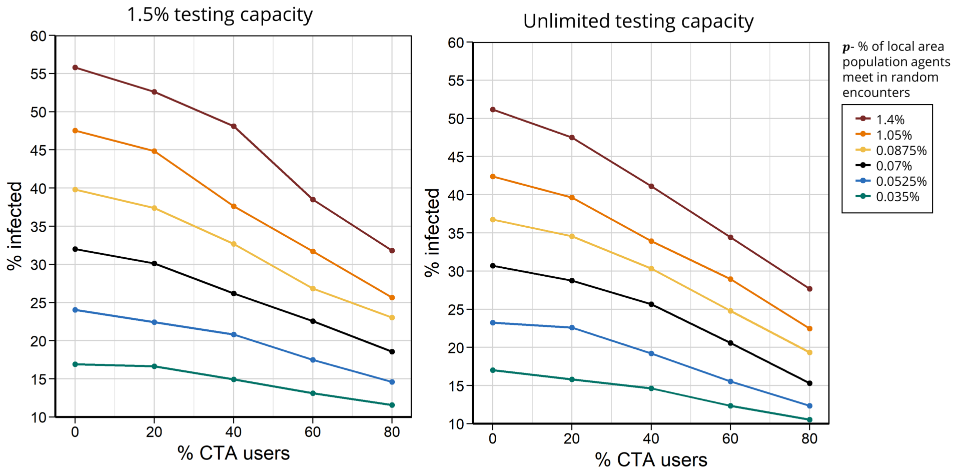

Figure 2: Sensitivity analysis for percentage of local area population that agents meet in random encounters (p). For each p value used in the simulation we present the percentage of the population infected during the course of the epidemic (y-axis) for varying rates of CTA users (x-axis). Values of p are presented by lines with unique colours. Black line represents the value used in the paper. The slope of the trajectory represent the influence of the CTA; a steeper negative slope represent a larger relative reduction in infections. Scenarios with testing capacity of 1.5% (left plot) and unlimited testing capacity (right plot).

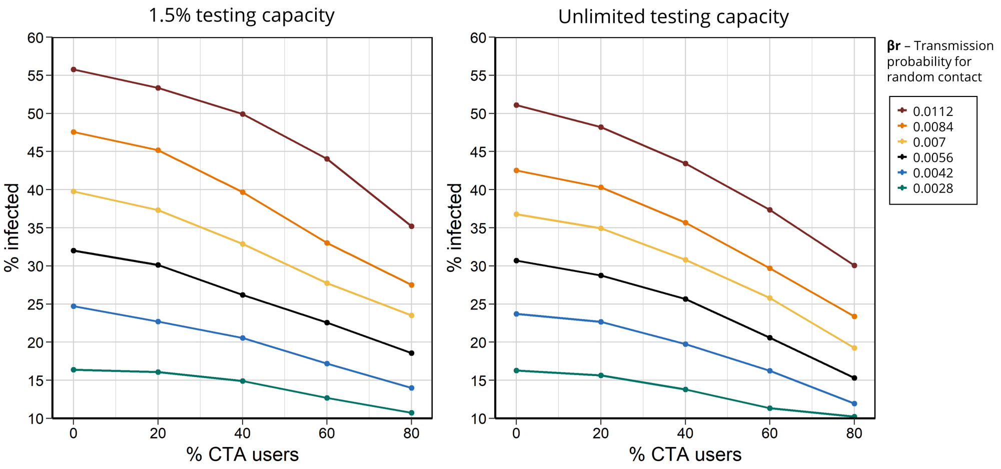

Figure 3: Sensitivity analysis for transmission probability per random contact (β_r). For each β_r value used in the simulation we present the percentage of the population infected during the course of the epidemic (y-axis) for varying rates of CTA users (x-axis). Values of β_r are presented by lines with unique colours. Black line represents the value used in paper. The slope of the trajectory represent the influence of the CTA; a steeper negative slope represent a larger relative reduction in infections. Scenarios with testing capacity of 1.5% (left plot) and unlimited testing capacity (right plot).

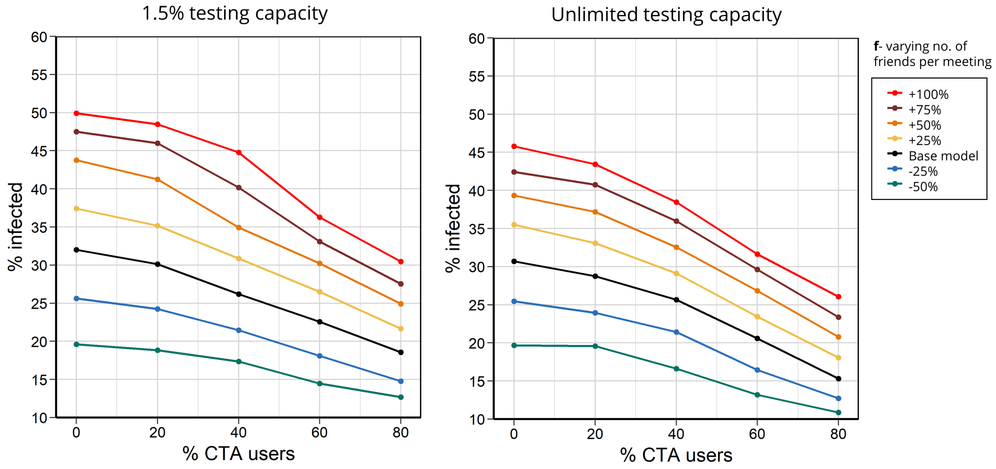

Figure 4: Sensitivity analysis for number of friends per social encounter (f). For each f value used in the simulation we present the percentage of the population infected during the course of the epidemic (y-axis) for varying rates of CTA users (x-axis). Values of f are presented by lines with unique colours. Black line represents the value used in the paper. The slope of the trajectory represent the influence of the CTA; a steeper negative slope represent a larger relative reduction in infections. Scenarios with testing capacity of 1.5% (left plot) and unlimited testing capacity (right plot).

Pseudo code of model step-function

The full source code and supporting datasets of the model are available at the following address: `https://github.com/harrykipper/covid` For ease of interpretation we offer a simplified version of the step function and the main infection process in the form of pseudocode, listed below.

```
function step():
tests-available = tests-available + tests-per-day
for (i in contacts_stored_in_CTAs) {
        days(i) = days(i) + 1
        if days(i) > 10 {remove_contact(i)}
        }

for (i in all_agents){
        if infected(i) {
                diseaseProgress()
                if asymptomatic(i) {b = b * Decay}
                if symptomsAppear {seek-testing}
```

```
                infect()
        }
        if crowd_worker(i){meet_customers}
}

function infect():
if isolating {b_hh = b_hh * 0.7}
for (i in household_members){
        if b_hh > random-float(1) {infect i}
        }

if not isolating {
        for (k in poisson (p * no_zone_residents)) {
                if cta_user(self) and cta_user(k)
                        {new_app_contact(self,k)}
                if (b_r * age_discount) > random-float(1)
                        {infect k}
        }
    if meeting-relatives{
       if b_c >random_float(1) {infect random-relative }
     }
        if office_worker {
                for (j in c_colleagues) {
                        if cta_user(self) and cta_user(j)
                                {new_app_contact(self,j)}
                        if b_c > random-float(1) {infect j}
                }
                if cta_user(self) and cta_user(random_other_colleague)
                        {new_app_contact(self,random_other_colleague)}
                if b_c > random-float(1) {infect random_other_colleague}
        } else if in_school {
                for (c in 1 to (no_of_classmates/2)){
                        if b_c * age_discount > random-float(1)
                                {infect c}
                        }
                }
        for (f in random(1,(no_of_friends * 0.1))){
                if cta_user(self) and cta_user(f)
                        {new_app_contact(self,f)}
                if b_c * age_discount > random-float(1) {infect f}
        }
}

function meet_customers():
for (k in poisson (3p * no_of_zone_residents)) {
        if cta_user(self) and cta_user(k){
```

```
                new_app_contact(self,k)
        }
        if infected() {
                if (b_r * age_discount) > random-float(1)
                        {infect k}
        } else {if infected(k) {
                if (b_r * age_discount) > random-float(1)
                        {infect self}
        }
}

function seek_testing():
if tests-available > 0 { get-tested() }
        else { decide_whether_to_isolate() }

function get-tested():
tests-available = tests-available - 1
if positive {
        for (i in household_members) {i.decide_whether_to_isolate()}
        for (i in relatives) {i.decide_whether_to_isolate()}
        if in_school {
                for (i in classmates) {i.isolate()}}
        if hasApp {
                for (i in app_contacts) {i.seek_testing()}
        }
}
```

References

[1] Davies, N. G, Klepac, P, Liu, Y, Prem, K, Jit, M, & Eggo, R. M. (2020) Age-dependent effects in the transmission and control of COVID-19 epidemics. *medRxiv* p. 2020.03.24.20043018.

[2] Verity, R, Okell, L. C, Dorigatti, I, Winskill, P, Whittaker, C, Imai, N, Cuomo-Dannenburg, G, Thompson, H, Walker, P. G. T, Fu, H, Dighe, A, Griffin, J. T, Baguelin, M, Bhatia, S, Boonyasiri, A, Cori, A, Cucunubá, Z, FitzJohn, R, Gaythorpe, K, Green, W, Hamlet, A, Hinsley, W, Laydon, D, Nedjati-Gilani, G, Riley, S, van Elsland, S, Volz, E, Wang, H, Wang, Y, Xi, X, Donnelly, C. A, Ghani, A. C, & Ferguson, N. M. (2020) Estimates of the severity of coronavirus disease 2019: a model-based analysis. *The Lancet Infectious Diseases* **20**, 669–677.

[3] Public Health England. (2020(accessed October 27, 2020)) Disparities in the risk and outcomes of covid-19 (`https://assets.publishing.service.gov.uk/government/uploads/system/uploads/attachment_data/file/892085/disparities_review.pdf`).

[4] Lauer, S. A, Grantz, K. H, Bi, Q, Jones, F. K, Zheng, Q, Meredith, H. R, Azman, A. S, Reich, N. G, & Lessler, J. (2020) The Incubation Period of Coronavirus Disease 2019 (COVID-19) From Publicly Reported Confirmed Cases: Estimation and Application. *Annals of Internal Medicine* **172**, 577–582.

[5] Chen, J, Qi, T, Liu, L, Ling, Y, Qian, Z, Li, T, Li, F, Xu, Q, Zhang, Y, Xu, S, Song, Z, Zeng, Y, Shen, Y, Shi, Y, Zhu, T, & Lu, H. (2020) Clinical progression of patients with COVID-19 in Shanghai, China. *Journal of Infection* **80**, e1–e6.

[6] Perez-Guzman, P. N, Daunt, A, Mukherjee, S, Crook, P, Forlano, R, Kont, M. D, Løchen, A, Vollmer, M, Middleton, P, Judge, R, Harlow, C, Soubieres, A, Cooke, G, White, P. J, Hallett, T. B, Aylin, P, Ferguson, N, Hauck, K, Thursz, M, & Nayagama, S. (2020) Report 17: Clinical characteristics and predictors of outcomes of hospitalised patients with COVID-19 in a London NHS Trust: a retrospective cohort study, (Imperial College Lodon), Technical report.
